# Impact of Transcendental Meditation on Left Ventricular Mass in African American Adolescents

**DOI:** 10.1155/2012/923153

**Published:** 2012-05-22

**Authors:** Vernon A. Barnes, Gaston K. Kapuku, Frank A. Treiber

**Affiliations:** ^1^Department of Pediatrics, Georgia Prevention Institute, Georgia Health Sciences University, HS1640, Augusta, GA 30912-3710, USA; ^2^Colleges of Nursing and Medicine, Medical University of South Carolina, Room 418, 99 Jonathan Lucas Street, Charleston, SC 29425, USA

## Abstract

*Background*. An early sign of ventricular remodeling is increased left ventricular mass (LVM) which over time may lead to left ventricular hypertrophy, the strongest predictor of cardiovascular morbidity and mortality, other than advancing age. *Methods*. 62 (30 TM; 32 CTL) African American adolescents (age 16.2 ± 1.3 years) with high normal systolic BP were randomly assigned to either 4-month Transcendental Meditation (TM) or health education control groups. The echocardiographic-derived measure of LVM index (LVMI = LVM/ht^2.7^) was measured before and after the 4-month TM study and at 4-month followup. 2D-guided M-mode echocardiography using a Hewlett Packard 5500 echosonograph was used to determine LVMI. *Results*. The TM group exhibited a greater decrease in LVMI at 4-month followup compared to the CTL group (−2.6 versus +0.3 gm/ht^2.7^, *P* < 0.04). The TM group exhibited a lesser increase in BMI at 4-month follow-up compared to the CTL group (0.2 ± 1.6 versus 1.1 ± 1.4, *P* < 0.03). *Conclusion*. These findings indicate that among a group of prehypertensive African American adolescents, 4 months of TM compared to heath education resulted in a significant decrease in LVMI, and these changes were maintained at 4-month follow-up.

## 1. Introduction

Increased left ventricular mass (LVM) has long been known to increase the risk for coronary artery disease (CAD), congestive heart failure, stroke, cardiac arrhythmias, and sudden death [1, 2]. Left ventricular mass may be reduced with BP reduction [3], and findings also suggest that lifestyle changes, such as moderate sodium restriction [4] as well as pharmacologic therapy [5], decrease LVM in both youth and adults. Longitudinal studies in sedentary subjects suggest exercise results in enlargement of LVM following training [6]. Prehypertension is associated with increased LVM in adolescents and young adults [7], and resting heart rate, systolic BP, gender, hemodynamic responses to stress, and adiposity were seen to be early determinants of LVM in children [8]. Other factors that can strain the workload on the heart are cardiovascular reactivity [9] and chronic stress (prolonged hyperactivation of the sympathetic nervous system) which favor increase in LVM [10].

 Studies have shown that Transcendental Meditation (TM) lowers indicators of psychosocial stress such as anger, hostility, and depression [11, 12]. TM has shown promise as a method for prevention and treatment of CVD and reducing CVD risk [13]. Among hypertensive patients, TM compared to standard treatment or health education results in greater reductions in systolic (SBP) and diastolic blood pressures (DBPs) [14, 15] and reduces CV mortality [16, 17]. In a prospective, single-blind, controlled study, Zamarra et al. reported that the TM program was useful in reducing exercise-induced myocardial ischemia in patients with coronary artery disease [18].Castillo-Richmond et al. found that over a period of six to nine months among a group of hypertensive African American (AA) adults, the TM program resulted in significant reduction in carotid intima-medial thickness (IMT) compared to a slight increase in the control group [19]. Another study of long-term TM practitioners showed acute reductions in BP during TM which were suggested to be a result of reductions in vasomotor tone (i.e., total peripheral resistance, a possible mechanism for BP reduction) [20]. These findings in adults have important implications for inclusion of TM in efforts to prevent and treat cardiovascular diseases and its clinical consequences [21] and have extended into the prehypertensive adolescent population, with TM decreasing resting SBP [22], ambulatory BP [23], and heart rate (HR) and cardiac output reactivity to behavioral stressors [22].

TM was shown to reduce LVM in a sample of hypertensive AA adults [24]. In another hypertensive AA sample (*N* = 102), TM practice did not change LVM after 7 months, whereas health education controls showed a significant increase, with a between-groups change score of 5.6 g/m^2^ [25]. These findings warrant further research to study the impact of meditation on LVM. To date, the impact of TM upon LVM in prehypertensive youth has not been examined. The present study examined the impact of TM on LVM in African American youth at increased risk for development of CVD. Collectively, based on the findings that TM beneficially lowers BP [23], we hypothesized that TM would decrease LVM.

## 2. Methods

### 2.1. Subjects

**Figure 1:**
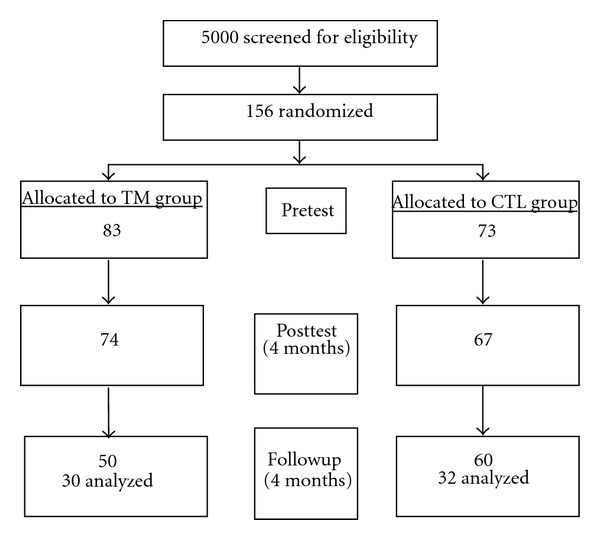
Flow of study recruitment.

 Voluntary BP screenings were conducted on approximately 5000 African American youth at five inner-city high schools in Augusta, GA. Parents were notified in advance of the health screening via a memo sent home by the school principal which resulted in a high rate of participation (99%). One hundred fifty-six subjects found to exhibit resting SBP in the ≥85th and ≤95th percentile for their age, sex, and height [26] on three consecutive days were invited to participate in the study on the basis of having high normal BP. Exclusion criteria included resting SBP >95th percentile, current involvement in a health promotion program, unwillingness to accept randomization into either study group, self-reported pregnancy, parental report of subject's history of congenital heart defect, diabetes, any chronic illness that required regular pharmacological intervention, or use of medications that may affect BP. 156 subjects were pretested following informed written consent and were randomly assigned to either 4-month TM or health education control (CTL) groups. The study was conducted over 4 years (eight 5-month semesters) with 4-month interventions (TM and CTL) each semester. Order of interventions was randomly counterbalanced between schools. 141 completed the posttest and 110 completed the 4-month follow-up evaluations (see Figure 1).

Data were missing for 48 subjects who had moved or otherwise could not be scheduled or due to technical difficulties (e.g., for 16 subjects, there were technical difficulties, that is, poor echocardiographic images, videotape problems, and subjects who could not be measured due to obesity) leaving 62 subjects for the analyses. Thirty TM (21 male subjects) and 32 CTL (24 male subjects) with data for all three visits were used in the final analyses.

### 2.2. Procedures

 All subjects abstained from exercise, smoking, and caffeinated beverages for 5 hours prior to testing. Height (with a stadiometer), weight (with a Detecto scale, Cardinal Scale Co, Webb City, MO), and skinfolds (triceps, subscapular and suprailiac crest, which served as surrogate indicators of changes in diet and/or physical activity) were recorded using established protocols [27] at pretest, posttest, and 4-month followup. Skinfolds were measured three times on the right side of the body with Lange calipers, and the readings were averaged. From these primary measures, the sum of the three skinfolds was calculated as a measure of body fat and body mass index (BMI; weight/height^2^) as a measure of general adiposity. After anthropometric measurements were obtained, supine resting hemodynamics were evaluated during minutes 10, 12, and 14. An appropriately sized blood pressure (BP) cuff was placed on the right arm for measurement of SBP, diastolic BP (DBP), and heart rate (HR) using a Dinamap Vital Signs Monitor (Model 1846SX, Critikon Incorporated, Tampa, FL).

 The echocardiographic-derived measure of LVM was obtained using 2D-guided M-mode echocardiography with a Hewlett Packard 5500. M-mode was used to determine interventricular septal thickness, LV cavity dimension, and LV free (posterior) wall thickness in triplicate in accordance with the American Society of Echocardiography (ASE) convention as described by Devereux et al. [28]. LVM was determined using the following validated formula: LVM = 0.8  [1.04 × ((IVS_d_+LVED_d_+LVPW_d_)^3^ − LVED_d_^3^)] + 0.6. LVM values were taken at the same time of day at pretest, posttest, and at 4-month follow-up. Technicians collecting the data were blinded as to study group affiliation. LVM was indexed to height^2.7^ (LVMI = LVM/ht^2.7^).

### 2.3. Interventions

 The TM technique is a simple mental procedure practiced for 15 minutes while sitting comfortably with eyes closed [29]. During the TM technique, it has been reported that the ordinary thinking process settles down, and a distinctive “wakeful hypometabolic state” is gained [30]. The format of instruction in the standard TM course includes introductory and preparatory sessions to outline the benefits and mechanics of the TM technique, a brief personal interview, a session of personal instruction, and three follow-up group sessions taking place over three consecutive days [31]. In the present study, after personal instruction, the TM group engaged in 15 min individual sessions at home and 15 min group sessions at school each school day, and 15 min twice daily individual home practice on weekends for 4 months. Daily group TM sessions were fitted into the school schedule without adversely impacting the academic schedule, for example, at the beginning of the school day during the “homeroom” period in a separate and secluded quiet location. Subjects were encouraged to continue daily home TM practice after the 4-month intervention was completed, but were not allowed to continue group sessions at school.

 The control (CTL) group was presented a 4-month didactic series of 15 min lifestyle education sessions each day based in part on National Institutes of Health guidelines on lowering BP through weight management, diet (increasing fruit and vegetable consumption and reducing caloric, fat and sodium intake), and increasing physical activity [26]. Identification of major sources of high-calorie foods and making appropriate substitutions were covered. Participants learned the value of mild-to-moderate intensity of daily physical activity. In order to control for bias, the same instructor was used for the CTL and TM group sessions. CTL group sessions were intended to provide instruction time and attention comparable to the TM group, but CTL group did not receive instructions for any stress reduction or relaxation techniques. Attendance was taken for all TM and CTL sessions at school, and self-report compliance records were kept for TM practice at home.

### 2.4. Data Analysis

 The statistical analysis was conducted on the SAS software package for analysis of variance and covariance (ANOVA/MANOVA) [32]. Differences between treatment groups in change in LVMI and other CV variables over the 8-month intervention period were assessed by MANCOVA using change scores in LVMI, covarying for pretest LVMI. The potential effects of SBP, DBP, HR, and BMI, on change in LVMI, were studied by entering them as covariates. Changes in secondary outcome variables, SBP, DBP, HR, and BMI, were assessed the same way as LVMI, that is, by ANCOVAs using the pretest levels of each variable as a covariate, and by paired *t*-tests to assess changes within groups. All statistical tests were two tailed.

## 3. Results

### 3.1. Anthropometric Measures

There were no statistical significant differences between treatment groups for preintervention anthropometric and hemodynamic parameters (all *P*s > 0.05, see Table 1). The TM group showed a trend for higher baseline heart rate (HR, *P* = 0.053). The compliance rate for twice daily practice in the TM group at home and at school was 77%.

### 3.2. Changes in LVM and LVMI

**Figure 2:**
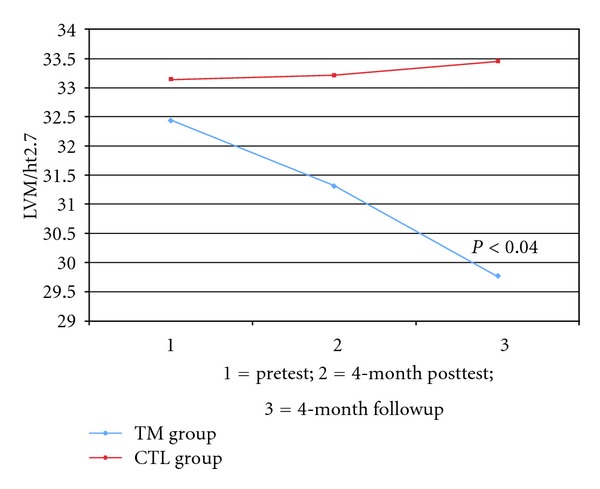
Changes in left ventricular mass index.

 The changes at posttest were not statistically significant. The TM group exhibited a greater decrease in LVM from baseline to the 4-month follow-up compared to the CTL group (−9.1 ± 20.2 versus 2.6 ± 22.7 gm, *P* < 0.04, see Table 2). The TM group exhibited a greater decrease in LVMI (LVM indexed by height^2.7^) from baseline to the 4-month follow-up compared to the CTL group (−2.7 ± 4.9 versus +0.3 ± 5.2 gm/ht^2.7^, *P* < 0.03, see Figure 2). Pearson correlations between change in LVMI and change in SBP were 0.16 for TM and 0.12 for CTL at posttest, and 0.02 for TM and 0.05 at follow-up (all *P* = ns).

### 3.3. Changes in BMI and Body Weight

 There were no significant differences between the groups in changes across the 8-month study for sum of three skinfolds (*P* > 0.05). The TM group exhibited a decrease in BMI at posttest compared and increase in the CTL group (–0.05 ± 1.0 versus 0.9 ± 1.2, *P* = 0.001, see Table 2). The TM group exhibited a lesser increase in body weight at the posttest compared to the CTL group (0.5 ± 3.0 versus 3.8 ± 4.5, *P* < 0.05). The TM group exhibited a lesser increase in BMI at 4-month follow-up compared to the CTL group (0.2 ± 1.6 versus 1.1 ± 1.4, *P* < 0.03, see Table 2). The TM group exhibited a lesser increase in body weight at the 4-month follow-up compared to the CTL group (1.5 ± 4.6 versus 3.2 ± 3.9, *P* = 0.004). Pearson correlations between change in LVMI and change in BMI were –0.08 for TM and 0.09 for CTL at posttest, and 0.10 for TM and 0.17 at follow-up (all *P* = ns).

## 4. Discussion

This study examined the impact of the TM technique on LVM in AA adolescents with high normal BP after the 4-month intervention and at 4-month followup. The TM group exhibited a significant decrease in LVM at the 4-month follow-up compared to the CTL group. This supports a previous finding with adult hypertensive AAs that showed an LVM decrease at 12 months in the TM group [24]. In addition, the TM group showed greater control of body weight, that is, blunted the expected normal body weight/BMI growth rate, after formal cessation of the intervention, and the difference between the two groups was significant at the 4-month followup.

The underlying physiological mechanisms responsible for LVMI reduction are not completely understood but may be related to BP reduction [23]. Stress reactivity has been associated with LVM increase two years later in adolescents [33]. Mental stress is associated with increased CV risk, because of the activation of sympathetic nervous system and the renin-angiotensin-aldosterone system. TM has been found to be associated with reduced BP reactivity to behavioral stress [22] related to reduced sympathetic nervous system tone [21]. TM has also been shown to have several beneficial hormonal and endocrine effects related to decreased sympathetic nervous system stimulation [34, 35] and decreased hypothalamic-pituitary-adrenocortical axis dysregulation [36, 37], as well as decreased cortisol levels [36], and diminished beta-adrenergic receptor sensitivity [38].

 The current findings should be interpreted cautiously due to the relatively small sample size. Loss to follow-up was not unexpected in this type of study, and this loss was similar for each group. Impact on the LV cavity or walls was not measured. The subjects were not screened for elevated LVM as a basis for study entry but rather were screened for elevated BP. Blinding of the sonographers to subjects' group classifications further decreased likelihood of any systematic bias in the measurements. The intervention was well received by the participants, and there were no adverse effects reported. Findings for BP changes [23] and beneficial impact upon measures of school behavior, that is, absenteeism, rule infractions, and suspension rates in the current study sample, have been published previously [39]. The TM group exhibited significantly greater decreases in ambulatory daytime systolic BP compared with little or no change in the CTL group across the 8-month study [23]. Future research should provide more precise information on changes in LV geometrical patterns linking BP and BMI [40].

## 5. Conclusion

To our knowledge, this is the first randomized, controlled study to demonstrate a decrease in LVM via a meditation program in prehypertensive adolescents. If this improvement is replicated among other at-risk groups and in cohorts of CVD patients, this will have important implications for inclusion of TM in the efforts to prevent and treat CVD and its clinical consequences. TM treatment may have therapeutic benefit for CVD patients and may impact favorably on prevention of vascular and myocardial complications of CVD. The decreases in LVM observed in this study, if maintained over time, have potentially important clinical significance. The successful implementation of the intervention points to the potential of school-based stress reduction programs as a means of decreasing likelihood of early onset of LVH in high-risk youth, particularly AAs.

## Figures and Tables

**Table 1:** Descriptive characteristics at pretest^a^.

	TM	CTL
	(*n* = 30; 9 F, 21 M)	(*n* = 32; 8F, 24 M)
Age (years)	15.7 ± 1.3	16.0 ± 1.3
Weight (kg)	79.3 ± 23.0	80.3 ± 21.1
Height (cm)	170.0 ± 9.2	171.9 ± 7.8
Body mass index (kg/m^2^)	27.09 ± 6.2	27.13 ± 6.9
SBP (mmHg)	128.0 ± 9.5	124.0 ± 12.7
DBP (mmHg)	64.7 ± 7.6	61.7 ± 9.4
HR (bpm)	69.6 ± 11.2	64.5 ± 9.1*
Sum of 3 skinfolds	55.72 ± 26.9	64.0 ± 35.3
LVM (gm)	137.7 ± 40.6	142.6 ± 37.2
LVMI	32.4 ± 7.4	33.2 ± 8.7

^a^Values are means ± standard deviations. *P value <0.06.

SBP = supine resting systolic blood pressure, DBP = supine resting diastolic blood pressure. HR = supine resting heart rate. LVM = left ventricular mass. LVMI = LVM indexed by height^2.7^.

**Table 2:** Comparison of changes from baseline to 4-month posttest and 4-month follow-up^b^.

	(4-month posttest)	(4-month follow-up)	(4-month posttest)	(4-month follow-up)	*P* value
	TM		CTL		
SBP	−4.7 ± 8.5	−2.7 ± 11.0	0.8 ± 8.0	−0.03 ± 11.9	0.37
DBP	−0.05 ± 7.8	0.08 ± 9.3	1.1 ± 6.4	1.6 ± 7.9	0.50
HR	−4.2 ± 10.8	−4.5 ± 10.6	−2.3 ± 6.0	−2.8 ± 6.6	0.44
LVM	−3.2 ± 20.2	−9.1 ± 20.2	1.4 ± 18.6	2.6 ± 22.7	0.036
LVMI	−1.1 ± 4.6	−2.7 ± 4.9	0.1 ± 4.3	0.3 ± 5.2	0.024
Weight	0.5 ± 3.0	1.5 ± 4.6	3.2 ± 3.9	3.8 ± 4.5	0.047
BMI	−0.1 ± 1.0	0.2 ± 1.6	0.9 ± 1.2	1.1 ± 1.4	0.028
Sum3SF	0.03 ± 0.2	0.04 ± 11.6	0.06 ± 0.3	−3.34 ± 12.3	0.435

^b^Change scores from baseline ± standard deviation. SBP = supine resting systolic blood pressure (mmHg), DBP = supine resting diastolic blood pressure (mmHg). HR = supine resting heart rate (bpm). LVM = left ventricular mass (gm). LVMI = LVM indexed by ht^2.7^. Weight (kg). BMI = body mass index. Sum 3SF = sum of three skinfolds.
